# A Rare Case of Gastric Outlet Obstruction Caused by a Duodenal Carcinoid

**DOI:** 10.7759/cureus.77729

**Published:** 2025-01-20

**Authors:** Caitlyn Cross, Angad Pordal

**Affiliations:** 1 General Surgery, Garden City Hospital, Garden City, USA

**Keywords:** anterior gastrotomy, carcinoid, en bloc surgical resection, gastric outlet obstruction, surgical enucleation

## Abstract

In this case report, we discuss a very rare presentation of a duodenal carcinoid tumor causing a gastric outlet obstruction that was initially misdiagnosed as gastroparesis due to uncontrolled diabetes. This tumor did not present with the usual symptoms or as carcinoid syndrome, as it was negative for all tumor markers and metanephrines. Treatment typically includes preoperative administration of somatostatin analogs, however, these were not used as the tumor showed no evidence of hormone secretion. Early operative treatment is indicated due to the poor prognosis associated with metastatic disease; however, this nonfunctioning tumor, with less than 3% Ki-67 positivity, was classified as low-grade.

During esophagogastroduodenoscopy (EGD) and surgical resection, the mass was observed to be highly mobile, intermittently prolapsing retrograde through the pylorus and acting as a one-way valve, obstructing the passage of fluids and food into the duodenum. Consequently, this gastric outlet-like picture was intermittent. The patient's history of uncontrolled diabetes and gastroparesis complicated the diagnosis as each episode of vomiting resolved on its own without any interventions. This made diagnosis of his underlying condition difficult. In this case, we present this rare type of carcinoid obstruction as well as surgical options and surveillance for these tumors.

## Introduction

Carcinoid tumors are rare, slow-growing tumors of ectodermal origin arising from enterochromaffin cells, most commonly presenting in the fifth to seventh decades of life. These tumors are hormonally active and are capable of secreting various neuroamines and peptides that can cause a variety of autonomic symptoms [1]. The most common location of these tumors is the small intestine, with 60% of all carcinoids occurring in the small bowel, followed by the tracheobronchial tree at 25%. Rarely, these tumors may present in the upper gastrointestinal tract with a duodenal presentation accounting for 2% of all gastrointestinal carcinoids [2]. While carcinoid tumors rarely present with symptoms, flushing, and watery diarrhea may occur. Gastrointestinal obstruction is a rare presenting symptom with very few previously published cases in the current literature. We present a rare case of gastric outlet obstruction, originally thought to be diabetic gastroparesis, secondary to a prolapsing duodenal carcinoid tumor that was managed operatively. 

## Case presentation

**Figure 1:**
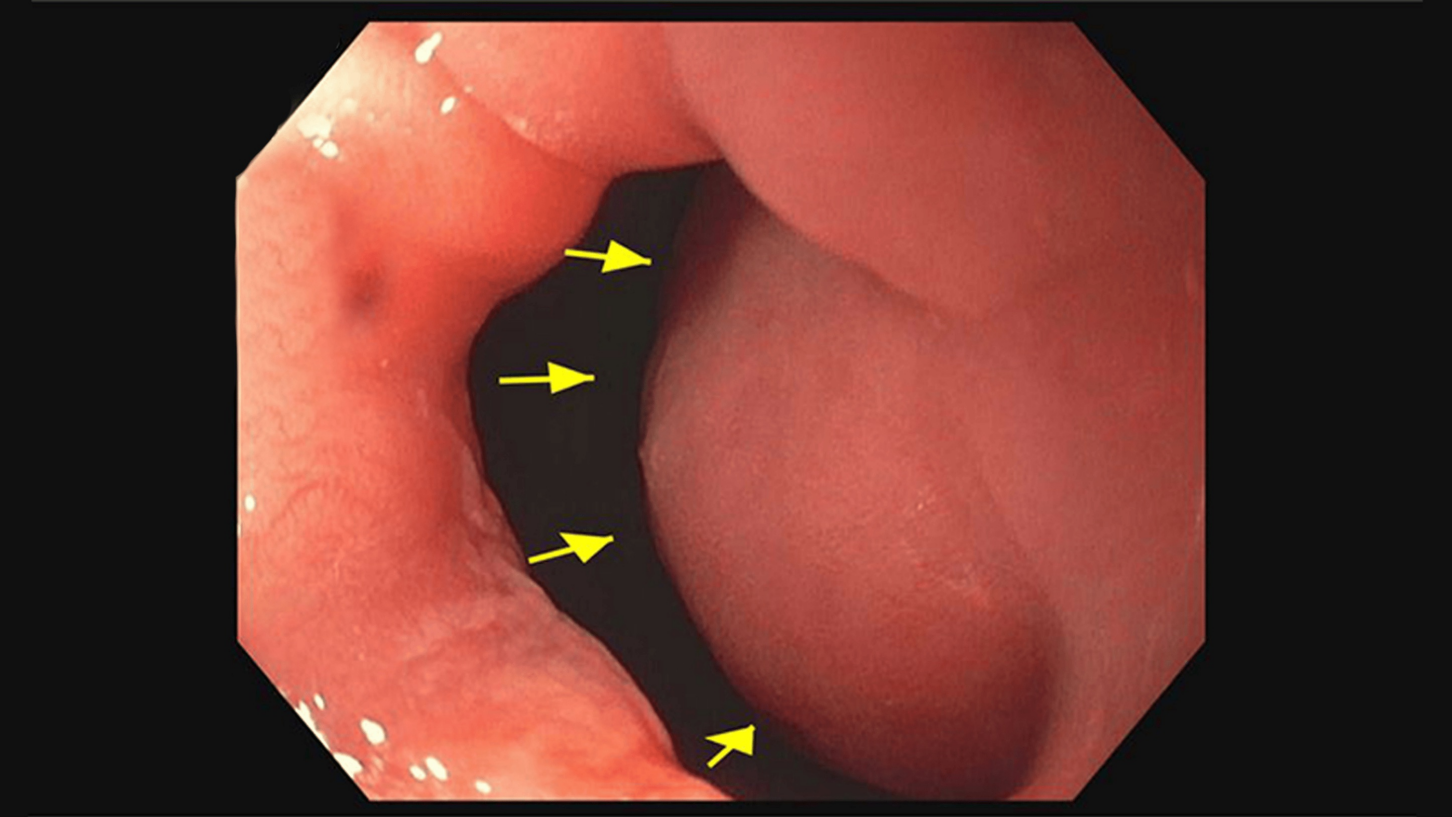
Esophagogastroduodenoscopy showing duodenal through the pylorus with near obstruction (75% of lumen)

**Figure 2:**
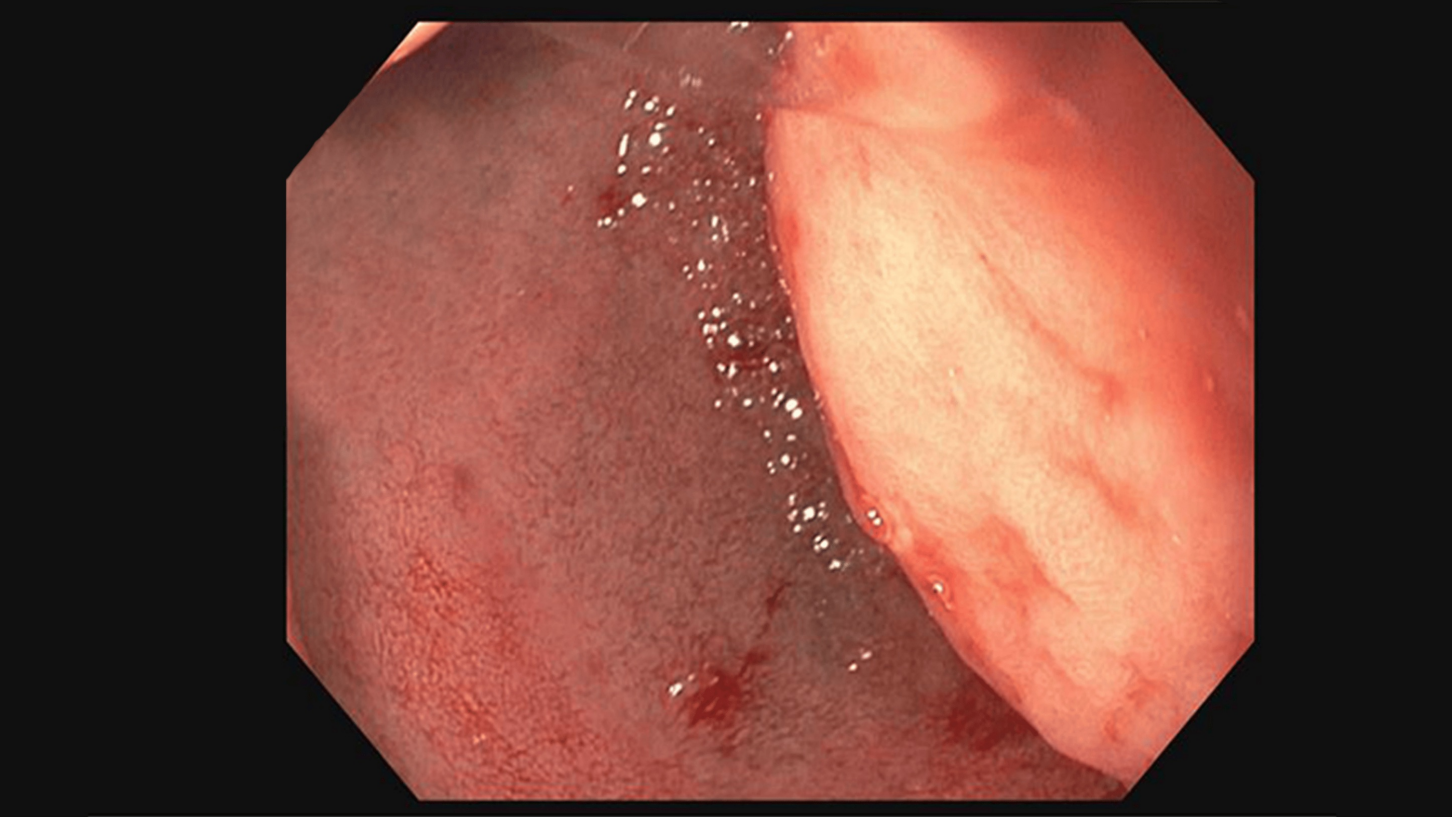
Esophagogastroduodenoscopy images showing mass shown on the right side of the duodenum within D1.

Our case involves a 42-year-old male with a past medical history significant for type 2 diabetes with the most recent hemoglobin A1C of 12.1. His diabetes was managed with lifestyle modifications and metformin and his asthma was treated with albuterol as needed. His prior gastroparesis was diagnosed several years ago but required no specific treatment. He presented with three months of intractable nausea and vomiting. The patient endorsed cyclic vomiting episodes which would last about three days. Each time, he was unable to keep down any solids or liquids. These episodes would resolve spontaneously and were not associated with any other symptoms such as abdominal pain, fever, or chills. There were no alleviating or aggravating factors. He presented to the emergency department on multiple occasions where it was clinically deemed that his symptoms were secondary to gastroparesis and each time was sent home with antiemetic therapy. He was originally diagnosed with gastroparesis five years ago after undergoing an esophagogastroduodenoscopy (EGD) and a nuclear gastric emptying study that showed 94% of food retained in the stomach one hour after ingestion. He again presented to our emergency department with his sixth episode of recurrent nausea and vomiting over the four months. At that time, gastroenterology was consulted. They proceeded with an EGD for further evaluation of these reoccurring symptoms, which was remarkable for a large post-pyloric proximal duodenal mass. This mass was highly mobile with evidence of intermittent obstruction of the antrum secondary to prolapsing of the mass through the pylorus. The mass obstructed approximately 75% of the pyloric junction, which increased to 100% during prolapse into the pylorus. At the time of the EGD, biopsies of the mass were taken; however, this mass was deemed not amendable to polypectomy or endoscopic mucosal resection (EMR) due to its size and location (Figures 1, 2). 

**Figure 3:**
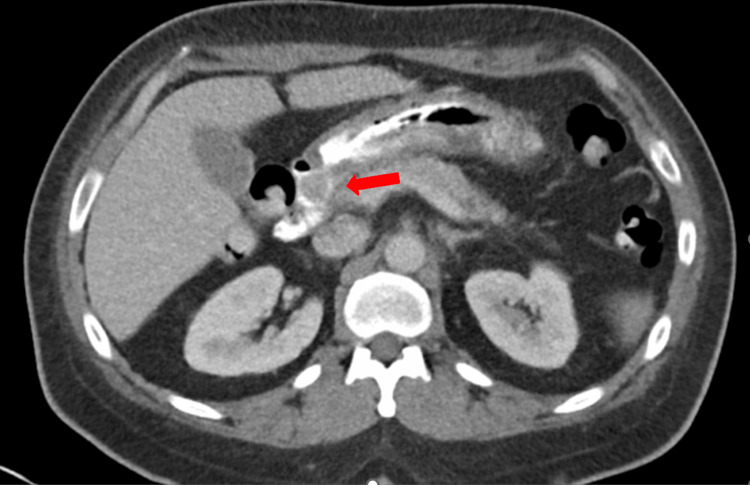
Computed tomography with oral and intravenous contrast showing duodenal mass in axial view without evidence of locoregional invasion

**Figure 4:**
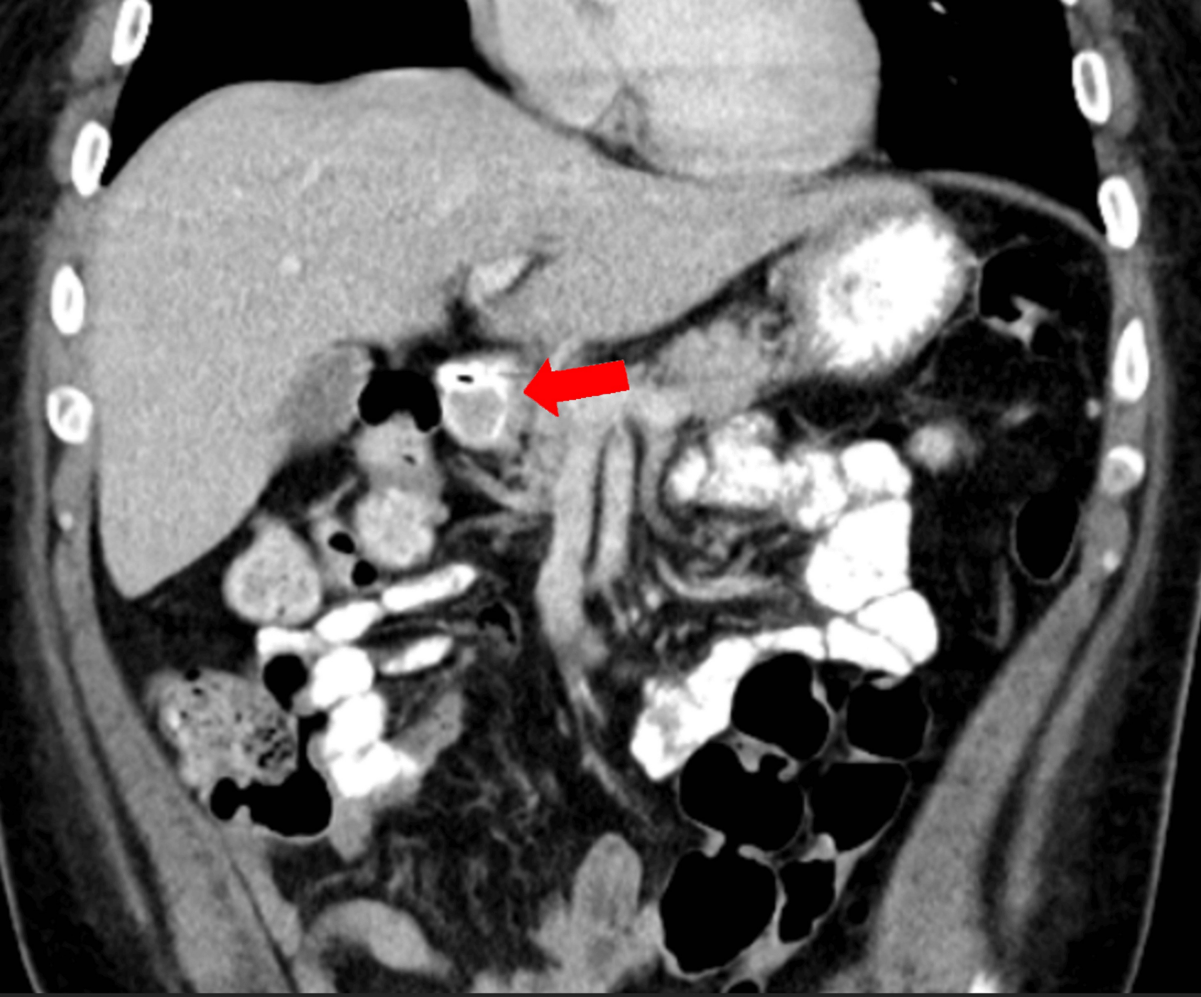
Computed tomography with oral and intravenous contrast showing duodenal mass in coronal view without evidence of locoregional invasion

Tumor markers were drawn, with results indicating normal serum chromogranin A of 88.7 ng/mL (0.0-101.8 ng/mL) and gastrin of 93 pg/mL (0-113 pg/mL). Urine and plasma metanephrines were also negative. Computed tomography (CT) abdomen and pelvis with contrast was then performed which was remarkable for a 1.5 x 1.6 x 1.5 cm intraluminal mass thought to be in the distal stomach versus proximal duodenum (Figures 3, 4). No extraluminal extension or evidence of metastatic disease was seen.

The patient was managed conservatively post EGD with a clear liquid diet and was subsequently discharged home on a liquid diet with follow-up in the general surgery clinic. Biopsy results showed well-differentiated carcinoid tumor staining positive for synaptophysin, chromogranin, and CD56, with less than 3% Ki-67 positive. The patient was subsequently seen in the general surgery clinic where he was counseled on treatment algorithms for carcinoid tumors ranging from enucleation of tumors measuring less than 2 cm up to the need for oncologic resection such as a Whipple procedure for larger tumors or tumors with unfavorable location near the Ampulla of Vater. The patient was counseled that their mass was less than 2 cm in diameter, well-differentiated, as well as mobile on endoscopic evaluation. Due to this, enucleation of the mass was recommended via anterior gastrotomy and/or pyloromyotomy. However, the patient was informed that pending actual operative or histologic findings, he may require a Whipple procedure for oncologic resection. The patient was agreeable to the enucleation of the carcinoid tumor and was subsequently scheduled for surgery. 

**Figure 5:**
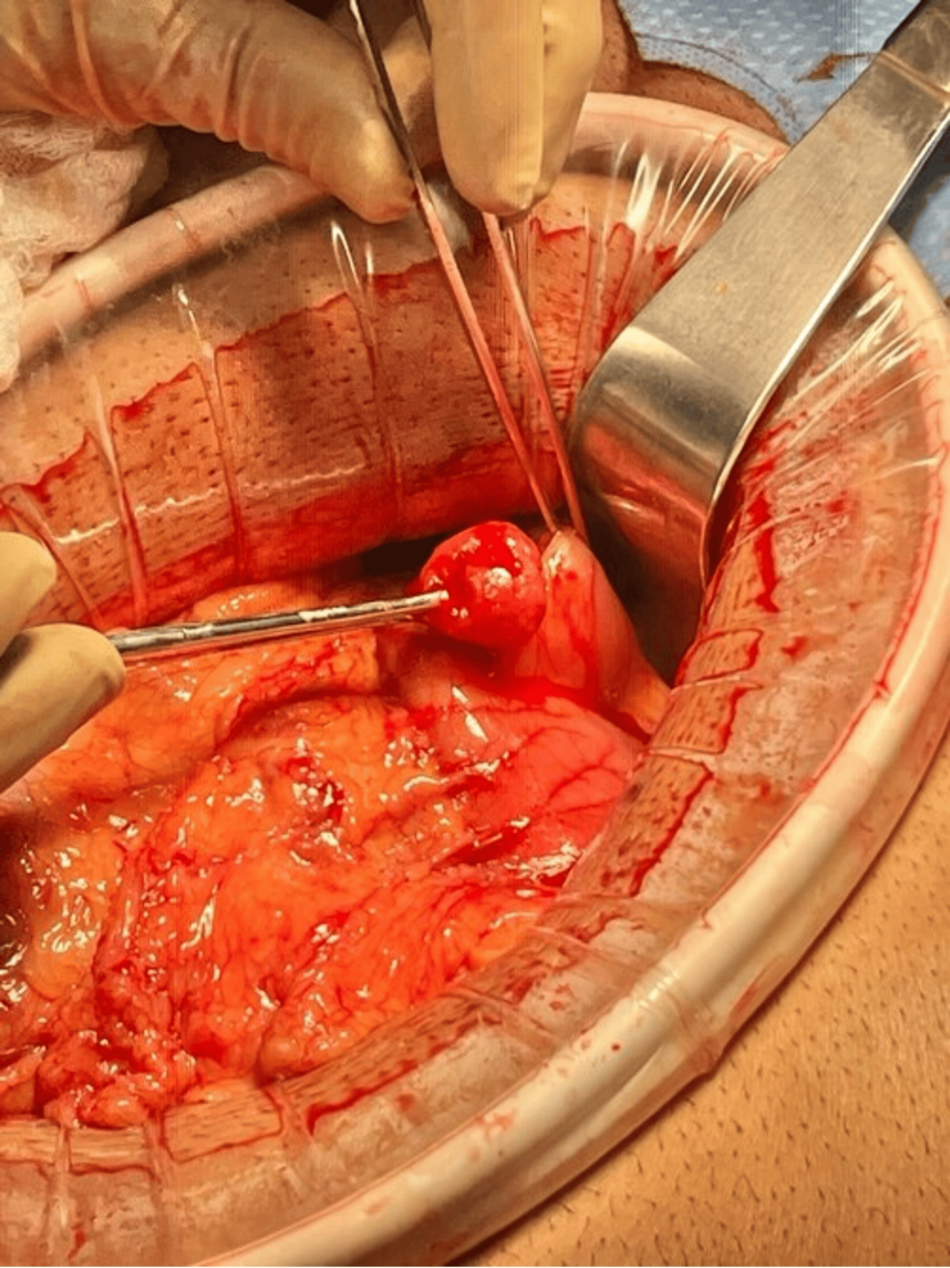
Intraoperative findings of duodenal carcinoid tumor expressed through pyloromyotomy incision.

**Figure 6:**
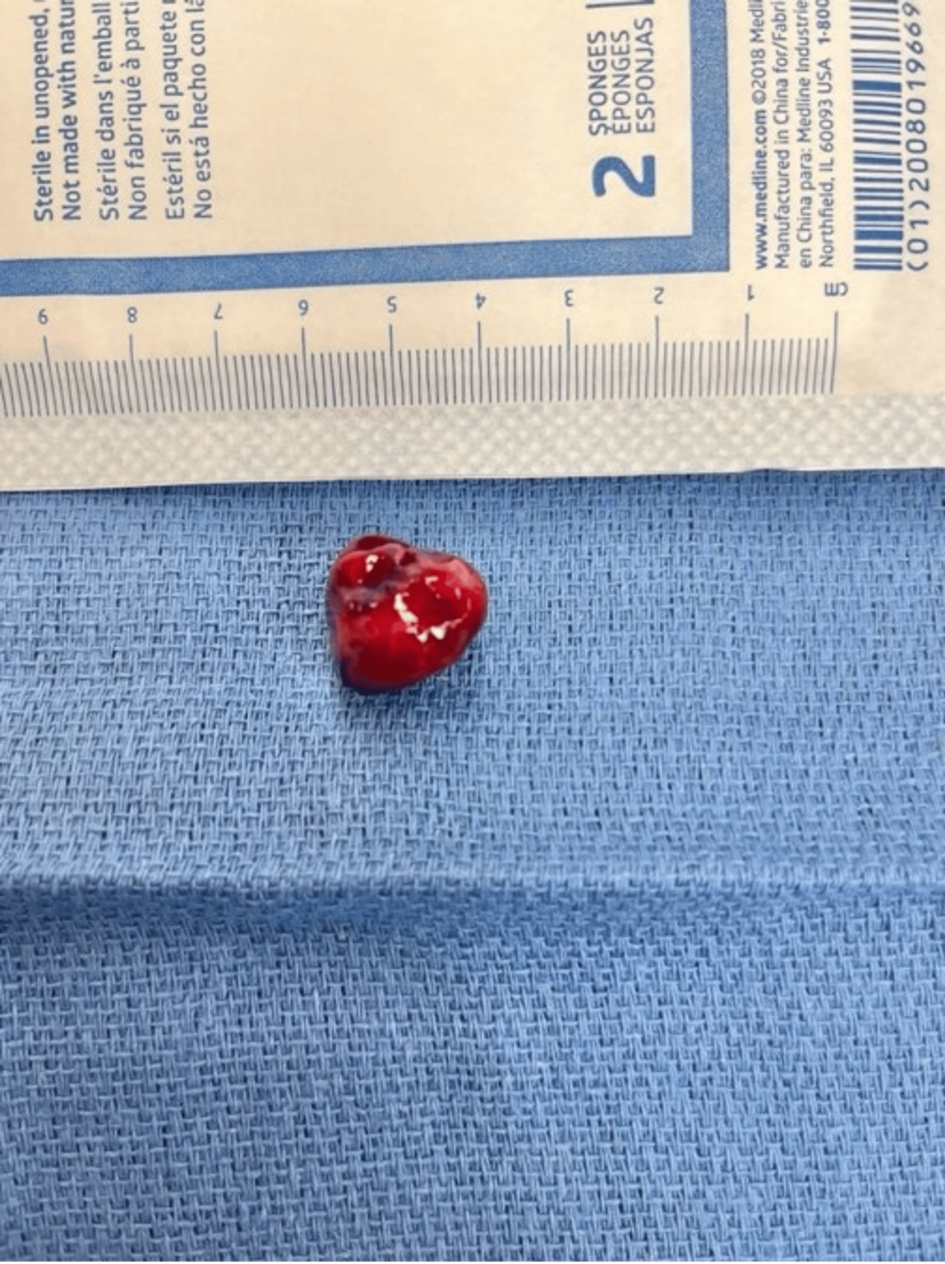
Mass after removal

**Figure 7:**
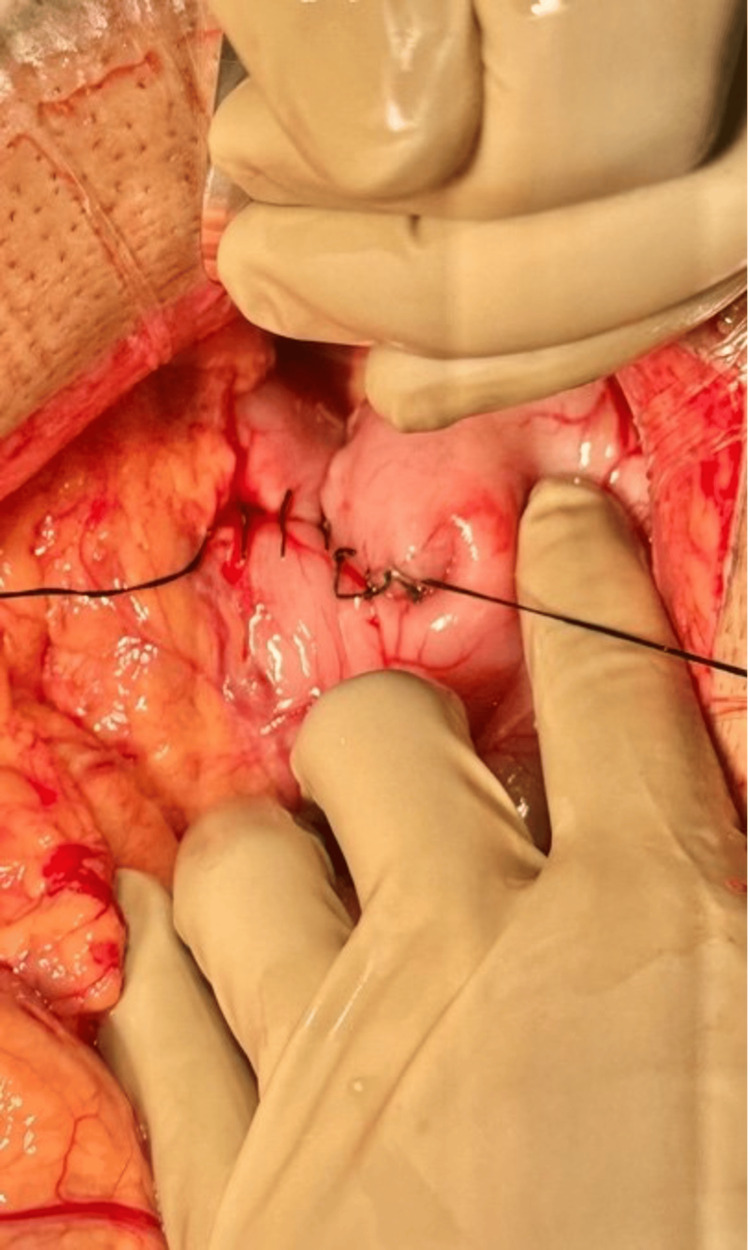
Transverse multi-layer closure of the enterotomy in Heineke–Mikulicz fashion.

The patient was placed in the supine position on the operating room table with both arms tucked for increased upper abdominal access. General anesthesia was induced via endotracheal intubation. Diagnostic laparoscopy then proceeded. No evidence of metastatic disease was seen. A firm mass was palpated with a grasper distal to the prepyloric vein of Mayo. The surgery was then converted to laparotomy in order to provide adequate exposure. Bovie electrocautery was used to create a transverse gastrotomy anterior and proximal to the pylorus. It was then extended longitudinally past the pylorus onto the first portion of the duodenum and the carcinoid tumor was easily delivered through this incision (Figure 5). The mass was removed en bloc by removing a portion of the mucosa of the duodenum with the attached underlying mass. This was sent to pathology for evaluation (Figure 6). The posterior mucosal defect was reapproximated using 2-0 Vicryl in a figure-of-eight fashion. The enterotomy was then closed in full-thickness Heineke-Mikulicz fashion, followed by the imbrication of the gastrotomy closure with 3-0 silk using a Lembert stitch pattern (Figure 7). The pyloroplasty closure was then submerged in saline and endoscopy was performed. The endoscope was advanced distal to the closure into the second portion of the duodenum. No leak or stricture was seen. A nasogastric (NG) tube was inserted. The abdomen was copiously irrigated, and a 19 French round channel Jackson-Pratt (JP) drain was inserted. A running looped polydioxanone (PDS) was used for fascial closure with skin staples for skin approximation. 

**Figure 8:**
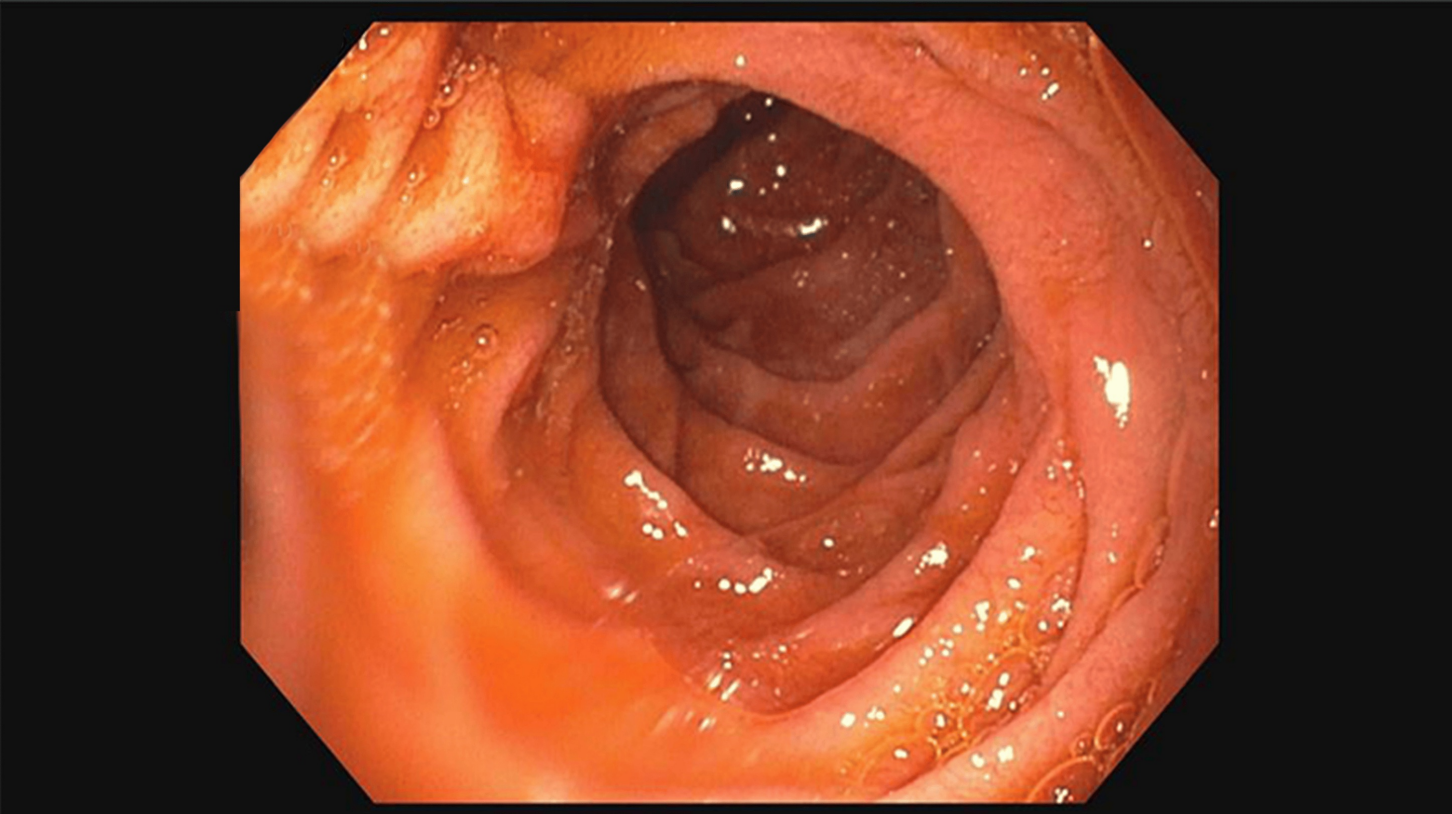
First portion of the duodenum post carcinoid resection showing no evidence of mucosal defect or recurrence.

**Figure 9:**
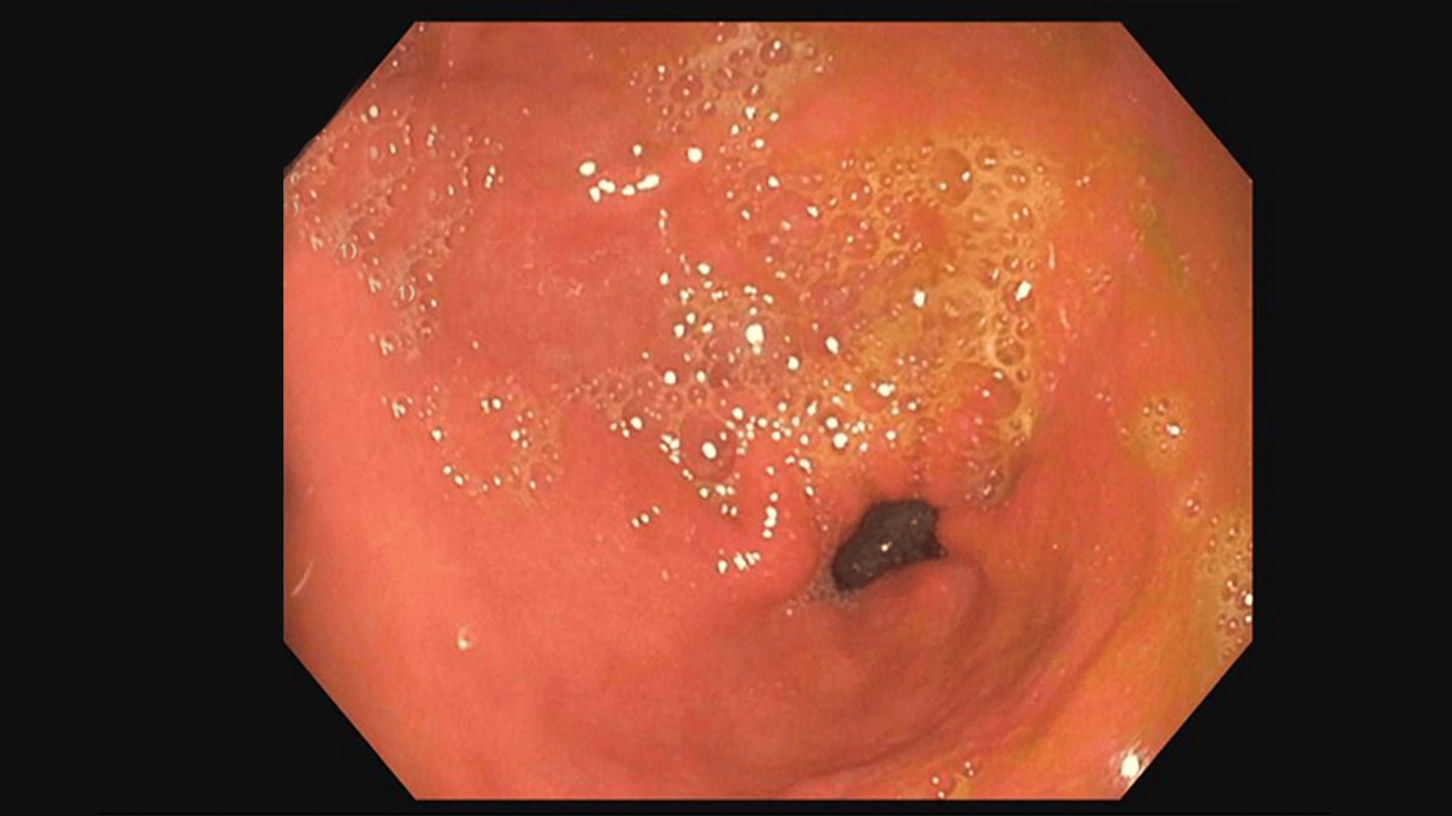
Pylorus post carcinoid resection

The NG tube was left in place and the patient was kept nothing by mouth (NPO) until postoperative Day 3. On postoperative Day 3, an upper gastrointestinal study was done with water-soluble gastrografin contrast which showed no leak. The NG tube was then removed and the diet was slowly advanced. He tolerated this well. He was discharged on postoperative Day 4 with a JP drain in place. Surgical pathology showed a well-differentiated carcinoid tumor invading the muscularis mucosa with negative margins. Again, the tumor stained positive for synaptophysin, chromogranin, and CD56, with less than 2% Ki-67 positive. He followed up one week after hospital discharge and was tolerating a regular diet without difficulty and the JP drain was removed in office. He followed up with hematology-oncology and had a positron emission tomography (PET) and gallium-68 dotatate (PET DOTATATE) scan which showed no evidence of hypermetabolic foci in the head, neck, chest, abdomen, or pelvis with a Krenning score of zero. He did not undergo this scan prior to surgical resection. Due to the lack of metastatic disease, as well as a low mitotic index, no adjuvant therapy was provided. The patient was subsequently seen in postoperative consultation at four weeks, as well as two months, and had no recurrent symptoms of nausea or emesis. A repeat EGD for evaluation of the surgical site was performed at two months and showed no evidence of stricture or recurrence (Figures 8, 9). 

## Discussion

Carcinoid tumors are a rare type of tumor with a prevalence of approximately 35 cases per 100,000. They most commonly present between the ages of 50 and 70, with an equal distribution between sexes. These tumors arise from neuroectodermal tissue and the cells arise from enterochromaffin cells or Kuchinsky cells which are located within the Crypts of Lieberkuhn within the gastrointestinal tract. These tumors are slow-growing and invade transmurally, spreading into the lymphatics and mesentery. Histopathology shows eosinophilic, round cells that are arranged in trabeculae [3]. These tumors commonly do not have symptoms as they tend to be discovered incidentally. When symptoms do present, they include flushing, diarrhea, and heart disease because these tumors secrete vasoactive peptides. These symptoms indicate metastatic disease within the liver as the liver is now unable to inactivate the vasoactive amines such as serotonin, 5-hydroxyindoleacetic acid (5-HIAA), serotonin, tachykinins, histamine, and prostaglandins. Carcinoid tumors are classified based on embryology and whether they originate from the foregut, midgut, or hindgut. These tumors are slow-growing and rarely cause mass effect-like symptoms because the autonomic symptoms are much more common. The diagnosis of these tumors is often delayed by up to seven years due to their slow-growing nature and vague complaints. The most common way these tumors are diagnosed is incidentally, during colonoscopy or endoscopy. Workup for these tumors includes imaging such as a CT scan or magnetic resonance imaging (MRI). More advanced imaging for these tumors includes a PET scan with DOTATOC and somatostatin receptor scintigraphy with Octreoscan, which are specific for somatostatin receptors on tumor cells. Lab studies for all patients with suspected carcinoid tumors include 24-hour urine with 5-hydroxyindoleacetic acid (5-HIAA), pancreastatin, and chromogranin-A [4]. Once the diagnosis is confirmed from a biopsy of accessible tumors and the above testing, the goal of treatment is to resect the primary tumor in its entirety and any distant nodal lesions that are accessible. Surgical resection depends on the location and size of the tumor as well as pathologic features that may predict the propensity for tumor spread [5]. 

Aside from size, the Ki-67 index is the most clinically important pathological feature dictating operative amenability. Previous studies have shown the Ki-67 index is significantly increased in those tumors that are atypical and have a higher propensity for metastasis. In addition, the Ki-67 index is a higher risk factor compared to lymphovascular invasion or other histological patterns. A Ki-67 index of 5% is often used as a cutoff with an index greater than 5% indicating a significantly increased preponderance for recurrence [4]. 

With an incidence of 0.3-0.9%, carcinoid tumors can be incidentally found in the appendix. If the carcinoid tumor is located at the tip of the appendix and is less than 2 cm, appendectomy is considered appropriate management. If, however, the tumor is 2 cm or greater or is located at the base of the appendix, an oncologic right hemicolectomy is indicated. Local excision can be performed for these tumors if they are less than 2 cm and located in the rectum. Endoscopic mucosal resection can be done for well-differentiated small tumors located within the stomach and duodenum. Due to the size and anatomic location of the carcinoid tumor presented in this case report, it was not amendable to endoscopic resection. For those patients with a large tumor, bowel resection or partial gastrectomy may be needed depending upon tumor location. For those tumors that are located within the distal first portion or second portion of the duodenum, a pancreaticoduodenectomy or Whipple procedure may be necessary. If a large tumor is located within the rectum, a low anterior resection may be necessary, while extensive invasive disease of the rectum may require abdominoperineal resection. [5] In patients with metastatic disease of the liver, options range from radiofrequency or ethanol ablation for small tumors to non-anatomic hepatic resections for larger tumors. Preoperative angioembolization of the hepatic artery to reduce blood flow to metastatic liver tumors greater than 5cm may also be considered [6].

As discussed above, the patient in this case was counseled on surgical options including the need for possible pancreaticoduodenectomy or Whipple procedure if the tumor was upstaged. In our patient, enucleation was possible due to the tumor size being close to but less than 2 cm diameter without distant metastasis or locoregional invasion. If total resection is achieved, five-year survival is as high as 90%. For those where only a partial resection of less than 90% of gross disease is achieved, somatostatin analogs should be used as an adjunct to decrease autonomic symptoms. Octreotide, as well as lanreotide, can be used for diarrhea as they both are agonists for somatostatin receptors. Loperamide can be used as an adjunct if diarrhea continues. Other advanced targeted therapies can also be considered including mTOR and interferon-alpha [6]. Radiotherapy targeting somatostatin analogs has been shown as an emerging treatment [4]. Surveillance for carcinoid tumor patients includes yearly upper or lower endoscopy and cross-sectional imaging depending on tumor location. In those whose tumor was less than 1 cm with no distant metastases, no surveillance is indicated [7]. 

The final pathology of the tumor revealed a well-differentiated carcinoid tumor invading the muscularis mucosa with the tumor staining positive for synaptophysin, chromogranin, and CD56, with less than 2% Ki-67 positive. While there are very few published cases of gastric outlet obstruction secondary to duodenal carcinoid, our pathology consistently aligns with previously published data. Another published case of gastric outlet obstruction secondary to a 2 cm duodenal carcinoid tumor published by Shaikh et al. yielded final pathology of duodenal carcinoid with submucosal involvement with chromogranin A and synaptophysin staining positively, similar to our patient [8]. 

## Conclusions

Carcinoid tumors are slow-growing tumors which cause vague or no symptoms in many patients. As a result, detection is often delayed by many years. In the case presented here, the patient had intermittent vomiting episodes due to the high mobile duodenal carcinoid tumor prolapsing through the pylorus and producing a gastric outlet obstruction. The intermittent nature of this condition delayed the diagnosis as the condition was initially thought to be diabetic gastroparesis. This case demonstrates the need for endoscopy, imaging, and lab work to appropriately characterize carcinoid tumors due to their indolent, slow-growing nature as well as surgical management options for removal of these tumors. This case report also discusses appropriate follow-up, screening, and monitoring for these tumors after they are diagnosed and surgically treated. 
